# Food Allergy Induces Anxiety-like Behavior and Neuroimmune Dysregulation in the Prefrontal Cortex of Mice

**DOI:** 10.3390/ijms27219431

**Published:** 2026-10-22

**Authors:** Yuchi Liu, Cheng Guo, Xiaodan Yu

**Affiliations:** Department of Developmental and Behavioral Pediatrics, Shanghai Children’s Medical Center, School of Medicine, Shanghai Jiao Tong University, Shanghai 200127, China; lyc_shsmu@sjtu.edu.cn (Y.L.); guochengc3@126.com (C.G.)

**Keywords:** food allergy, anxiety-like behavior, neuroinflammation, microglia, transcriptomics, Akt signaling

## Abstract

Food allergy (FA) is associated with anxiety, but the underlying mechanisms remain incompletely understood. An ovalbumin (OVA)-induced FA model was established in female BALB/c mice to evaluate anxiety-like behavior and characterize molecular alterations in the prefrontal cortex (PFC). We assessed the FA model by anaphylactic scores, OVA-specific IgE and IgG1 levels, serum cytokines, and intestinal histology, and evaluated anxiety-like behavior using the open field test (OFT) and elevated plus maze (EPM). Mice with OVA-induced food allergy exhibited pronounced allergic responses and intestinal mucosal injury, together with reduced center-zone exploration in the OFT and reduced open-arm exploration in the EPM. In the PFC, inflammatory and chemotactic mediators were increased, accompanied by enlarged Iba1-positive microglial soma area. Bulk RNA sequencing identified 183 differentially expressed genes, including upregulated genes involved in immune and inflammatory responses and downregulated genes associated with neuronal activity and plasticity. Western blotting further showed an increased p-Akt (Ser473)/Akt ratio. These findings reveal that OVA-induced food allergy in female BALB/c mice is associated with neuroimmune dysregulation and broader transcriptional alterations in the PFC involving immune and inflammatory responses and processes related to neuronal activity and plasticity, and suggest that altered Akt-related signaling may provide a molecular link between these changes.

## 1. Introduction

Food allergy (FA) is an adverse reaction to food mediated by the immune system and can manifest in multiple organ systems, including the skin, respiratory tract, and gastrointestinal tract. The prevalence of food allergy has increased worldwide, adversely affecting the quality of life of patients and their families [1,2]. Previous research has focused predominantly on peripheral pathological processes, including intestinal injury, mast cell activation, and T helper 2 (Th2)-type immune responses [3,4,5]. As recognition of the systemic effects of food allergy grows, its potential impact on the central nervous system and psychological states has also attracted increasing attention [6,7]. Emerging evidence suggests that food allergy may affect not only peripheral immunity and gastrointestinal function but also emotional and behavioral states [8,9,10,11], with anxiety receiving increasing attention.

Accumulating evidence indicates that anxiety may represent an important emotional phenotype associated with food allergy. A longitudinal cohort study reported that food allergy was associated with higher rates of mother-reported depression, anxiety, and attention-deficit/hyperactivity disorder during adolescence [8]. Another longitudinal study found associations between food allergy and symptoms of separation anxiety, generalized anxiety, and attention-related difficulties, as well as subsequent increases in anxiety and depressive symptoms, although the prevalence of diagnosed psychiatric disorders was not higher [9]. Additionally, early food sensitization has been investigated in relation to infant neurodevelopment, with sex-dependent associations reported for social–emotional developmental outcomes [6]. In animal studies, food allergy has likewise been associated with anxiety-like behavior and increased c-Fos expression in brain regions related to anxiety [12,13], providing experimental support for food allergy-associated anxiety-like behavior. However, the underlying neurobiological mechanisms remain insufficiently understood.

The prefrontal cortex (PFC) is a key brain region involved in emotion regulation, threat appraisal, and anxiety-related behavior [14,15,16], and its local neuroimmune homeostasis is closely linked to normal emotional function [17,18]. Previous work has shown that food-allergic mice exhibit anxiety-like behavior, accompanied by PFC inflammation and microglial activation [11], suggesting that food allergy-associated emotional abnormalities may be related to PFC neuroimmune dysregulation. Similarly, a study using an acute sleep-deprivation model reported that PFC microglia and microglia-associated neuroinflammation contribute to the development of anxiety-like behavior [18]. However, the molecular mechanisms underlying food allergy-associated anxiety-like behavior and PFC neuroimmune dysregulation remain incompletely understood, particularly the broader transcriptomic alterations involving immune and neuronal functional programs and the signaling pathways associated with these changes.

Accordingly, this study used an OVA-induced food allergy mouse model to systematically evaluate the effects of food allergy on peripheral immune responses, anxiety-like behavior, and alterations in the PFC neuroimmune microenvironment. We performed PFC cytokine assays, microglial immunofluorescence, transcriptomic sequencing, and Western blot analysis to characterize broader molecular alterations in the PFC and explore signaling pathways potentially involved in these changes. This study aimed to provide a broader molecular characterization of PFC alterations associated with food allergy and to improve understanding of the central nervous system consequences of food allergy.

## 2. Results

### 2.1. Establishment of an OVA-Induced Food Allergy Model

**Figure 1:**
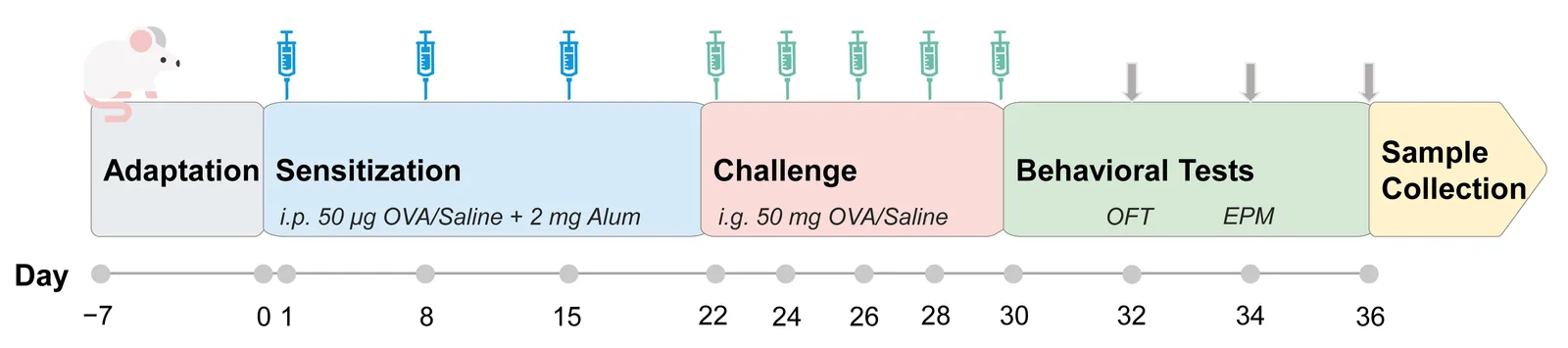
Experimental design for the OVA-induced food allergy model in mice. Following 1 week of acclimatization, mice underwent sensitization and challenge phases. The OVA group received intraperitoneal injections of OVA with aluminum adjuvant on Days 1, 8, and 15, followed by oral OVA challenges on Days 22, 24, 26, 28, and 30. The CON group received corresponding saline treatments. Behavioral testing was subsequently performed using the open field test (OFT) on Day 32 and the elevated plus maze (EPM) on Day 34. Blood and tissue samples were collected on Day 36. OVA, ovalbumin; i.p., intraperitoneal; i.g., intragastric; OFT, open field test; EPM, elevated plus maze.

**Figure 2:**
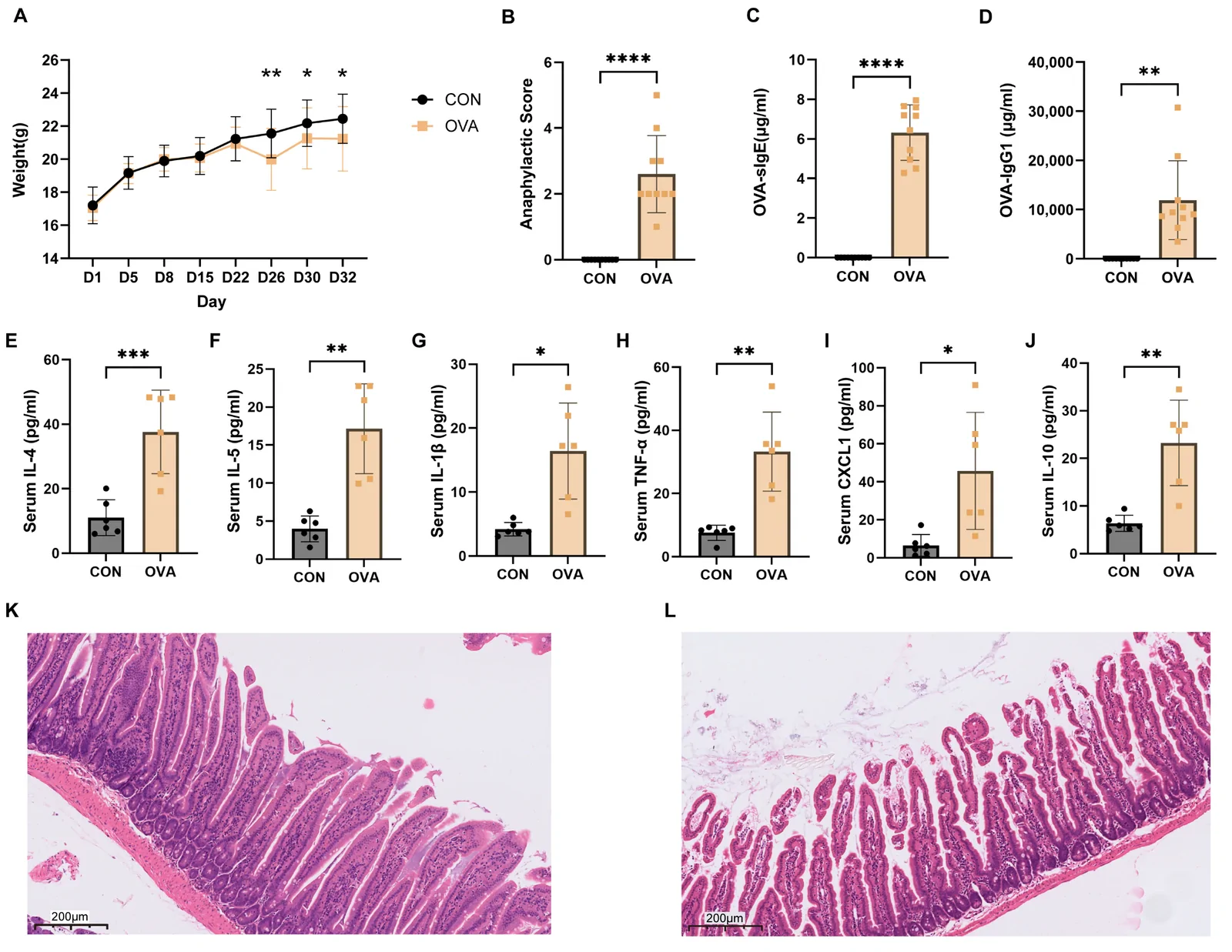
OVA-induced food allergy induces systemic allergic and inflammatory responses and intestinal mucosal injury. (**A**) Body weight changes during the experimental period. (**B**) Anaphylactic scores after the final OVA challenge. (**C**,**D**) Serum OVA-specific IgE and IgG1 levels, respectively. (**E**–**J**) Serum levels of IL-4, IL-5, IL-1β, TNF-α, CXCL1, and IL-10, respectively. (**K**,**L**) Representative hematoxylin and eosin (H&E)-stained images of the small intestine from the CON and OVA groups, respectively. *n* = 21/group for body-weight, *n* = 10/group for anaphylactic score analysis and OVA-specific IgE and IgG1 measurements, and *n* = 6/group for serum cytokine analyses. Data are presented as mean ± SD. * *p* < 0.05, ** *p* < 0.01, *** *p* < 0.001, **** *p* < 0.0001. CON, control; OVA, ovalbumin.

As shown in Figure 1, an OVA-induced food allergy model was successfully established by repeated oral challenge. During the challenge phase, body-weight gain was markedly slower in the OVA group than in the CON group (Figure 2A). After the final challenge, anaphylactic scores were assessed. The OVA group showed significantly higher anaphylactic scores than the CON group (Figure 2B).

Serum measurements showed significantly increased levels of OVA-specific IgE and IgG1 in the OVA group compared with the CON group (Figure 2C,D), indicating a pronounced antigen-specific sensitization response following repeated OVA exposure. To further assess systemic immune responses, multiple serum cytokines were measured (Figure 2E–J). IL-4 and IL-5 were significantly elevated in the OVA group, consistent with a prominent Th2-type immune response. The pro-inflammatory cytokines IL-1β and TNF-α were also significantly elevated in the OVA group, indicating systemic inflammation. In addition, CXCL1 levels were significantly elevated in the OVA group, suggesting enhanced chemotaxis. IL-10 was also significantly increased in the OVA group, suggesting a concomitant immunoregulatory response. Overall, the serum cytokine results showed a prominent Th2-type immune response together with enhanced pro-inflammatory and chemotactic responses.

Hematoxylin and eosin (H&E) staining was performed to assess the effects of OVA-induced food allergy on intestinal morphology. The intestinal mucosa remained intact in CON mice, with densely and regularly arranged villi, whereas OVA mice exhibited marked structural injury, including disruption of the villus surface and mucosal damage (Figure 2K,L). Taken together, repeated oral OVA challenge induced a robust allergic phenotype, antigen-specific immune response, and systemic inflammation accompanied by intestinal mucosal injury, confirming successful establishment of the OVA-induced food allergy model.

### 2.2. OVA-Induced Food Allergy Is Associated with Anxiety-like Behavior

**Figure 3:**
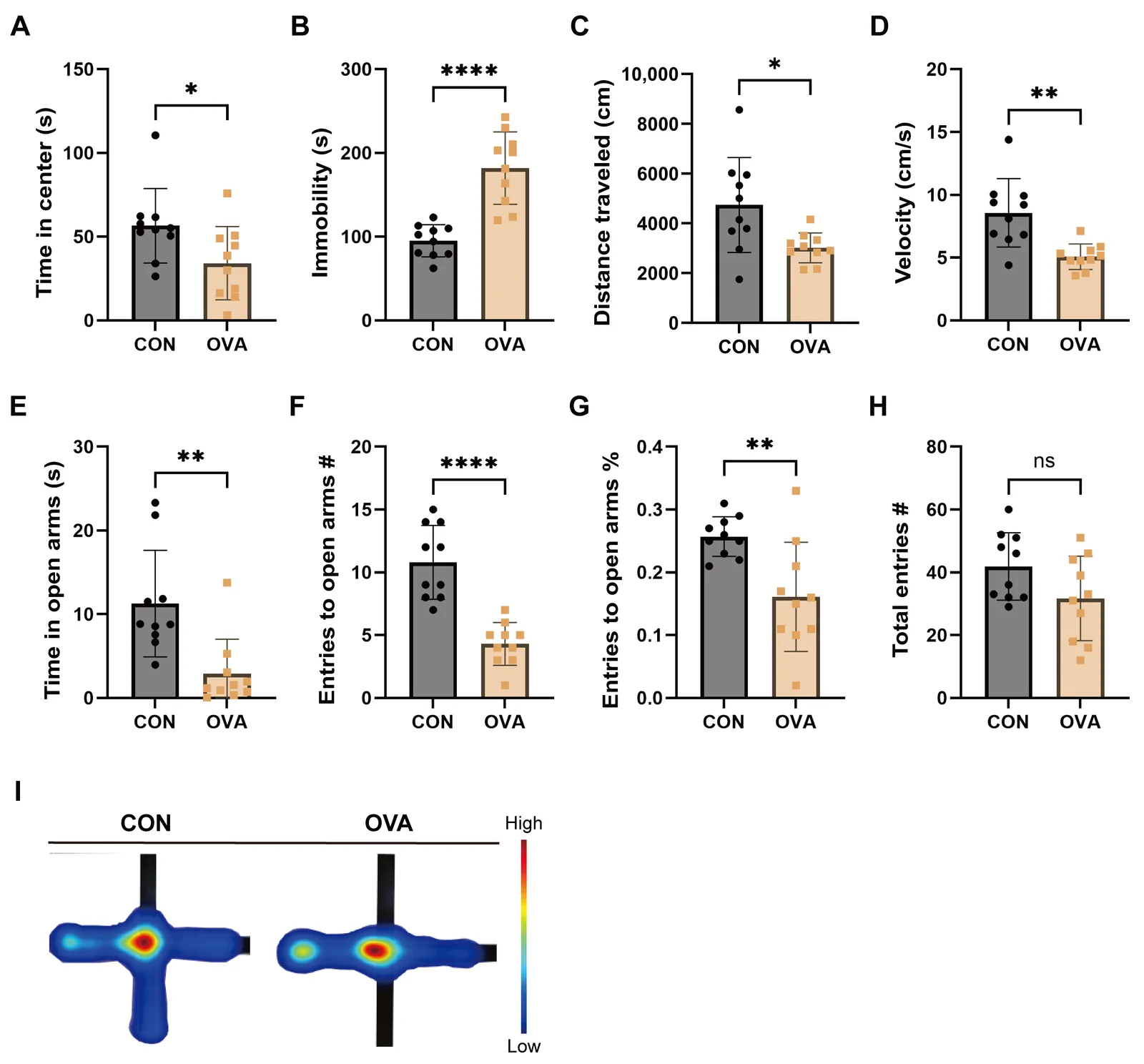
OVA-induced food allergy is associated with anxiety-like behavioral abnormalities in mice. (**A**–**D**) Open field test (OFT). (**A**) Center-zone time, (**B**) immobility time, (**C**) total distance traveled, and (**D**) velocity. (**E–H**) Elevated plus maze (EPM). (**E**) Open-arm time, (**F**) open-arm entries, (**G**) open-arm entry percentage, and (**H**) total arm entries. (**I**) Representative EPM movement trajectory heatmaps. *n* = 10/group. Data are presented as mean ± SD. * *p* < 0.05, ** *p* < 0.01, **** *p* < 0.0001; ns, not significant. CON, control; OVA, ovalbumin.

To evaluate the effects of food allergy on emotional behavior, the open field test (OFT) was first used to assess spontaneous locomotor and exploratory activity. Compared with CON mice, OVA mice showed significantly reduced center-zone time and increased immobility time (Figure 3A,B). Total distance traveled and velocity were also significantly reduced (Figure 3C,D), indicating suppressed spontaneous activity and exploration under food-allergic conditions. Together with reduced center-zone activity, these findings indicate increased center-zone avoidance and suggest anxiety-like behaviors.

To further validate the anxiety-like phenotype and assess whether it was influenced by reduced overall locomotor activity, the elevated plus maze (EPM) was subsequently performed. Compared with CON mice, OVA mice showed significantly shorter open-arm time as well as fewer open-arm entries and a lower open-arm entry percentage (Figure 3E–G). Notably, total arm entries did not differ significantly between groups (Figure 3H), suggesting that reduced open-arm exploration could not be fully attributed to a general reduction in exploratory activity. Representative EPM trajectory heatmaps further showed more activity in the open-arm regions in CON mice, whereas OVA mice were predominantly active in the central and closed-arm regions (Figure 3I), further indicating avoidance of open and exposed areas.

Taken together, OVA-induced food allergy reduced spontaneous locomotor and exploratory activity and produced consistent open-area avoidance across two distinct behavioral paradigms, supporting the presence of anxiety-like behaviors.

### 2.3. OVA-Induced Food Allergy Elicits PFC Inflammation and Microglial Morphological Alterations

**Figure 4:**
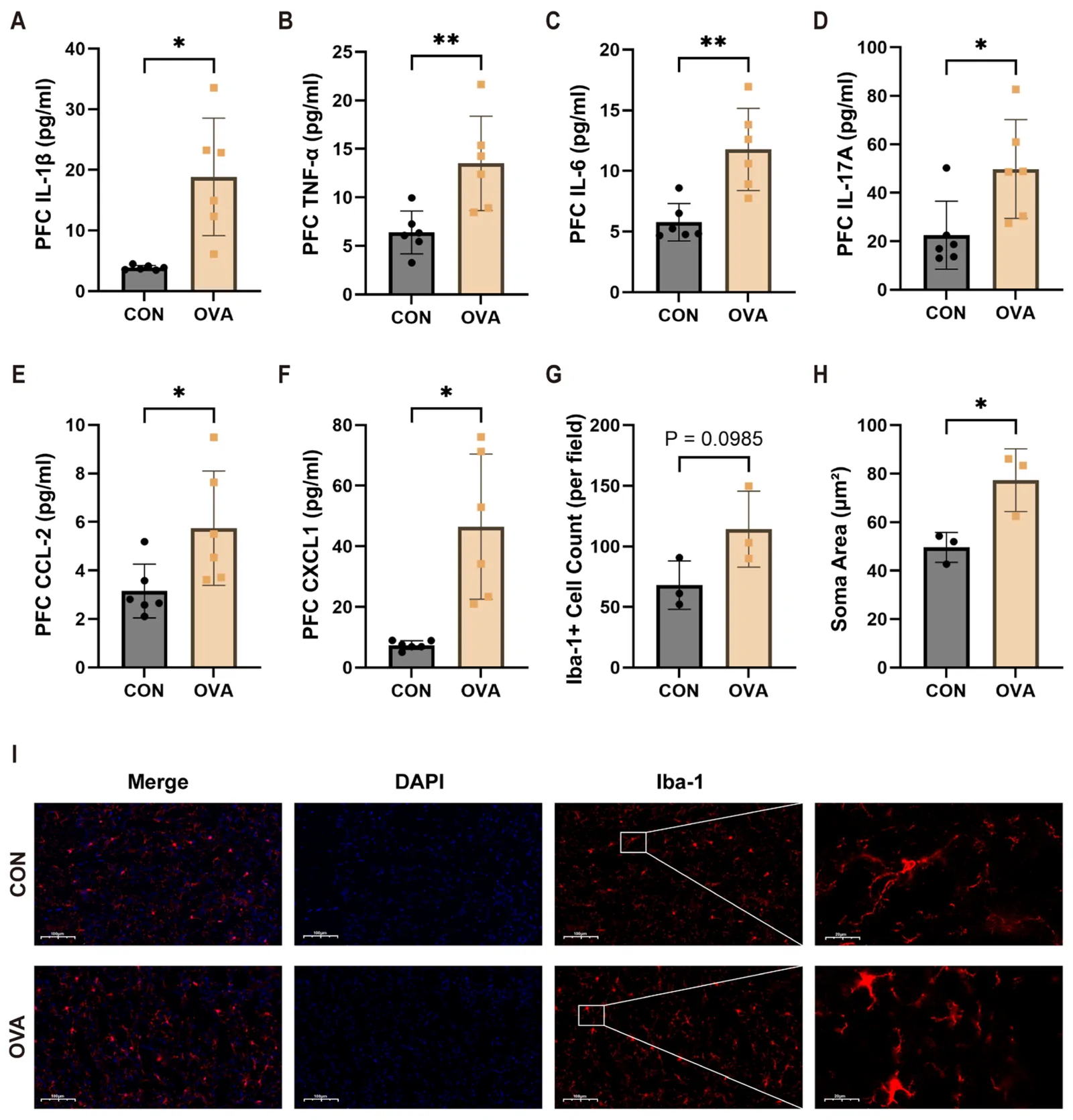
OVA-induced food allergy is associated with PFC inflammation and microglial morphological changes. (**A**–**F**) Levels of IL-1β, TNF-α, IL-6, IL-17A, CCL2, and CXCL1 in PFC tissue, respectively. (**G**) Quantification of Iba1-positive microglia in the PFC. (**H**) Microglial soma area. (**I**) Representative Iba1 immunofluorescence images of the PFC. *n* = 6/group for cytokine analyses and *n* = 3/group for immunofluorescence analyses. Data are presented as mean ± SD. * *p* < 0.05, ** *p* < 0.01; ns, not significant. Scale bars = 100 μm for low-magnification images and 20 μm for enlarged images. CON, control; OVA, ovalbumin. Iba-1, ionized calcium-binding adaptor molecule 1; PFC, prefrontal cortex.

Because the PFC is an important brain region involved in the regulation of emotion and anxiety-related behavior [14,15], we next examined neuroimmune changes in this region under food-allergic conditions. Compared with CON mice, OVA mice showed significantly increased levels of IL-1β, TNF-α, IL-6, and IL-17A in the PFC (Figure 4A–D), indicating enhanced local pro-inflammatory immune responses. PFC levels of CCL2 and CXCL1 were also significantly increased (Figure 4E,F), suggesting enhanced inflammation-associated chemotactic signaling.

Microglia are key immune effector cells in the central nervous system and play important roles in neuroinflammation and regulation of the neural microenvironment [19]. Iba1 immunofluorescence was therefore performed to assess PFC microglia (Figure 4I). The number of Iba1-positive microglia showed an increasing trend in OVA mice but did not reach statistical significance (Figure 4G). In contrast, microglial soma area was significantly increased in OVA mice (Figure 4H), indicating activation-related morphological remodeling.

Overall, OVA-induced food allergy enhanced local inflammatory and chemotactic signaling in the PFC and was accompanied by marked microglial morphological remodeling, consistent with substantial alterations in the PFC neuroimmune microenvironment.

### 2.4. Transcriptomic Remodeling of the PFC in OVA-Induced Food Allergy

**Figure 5:**
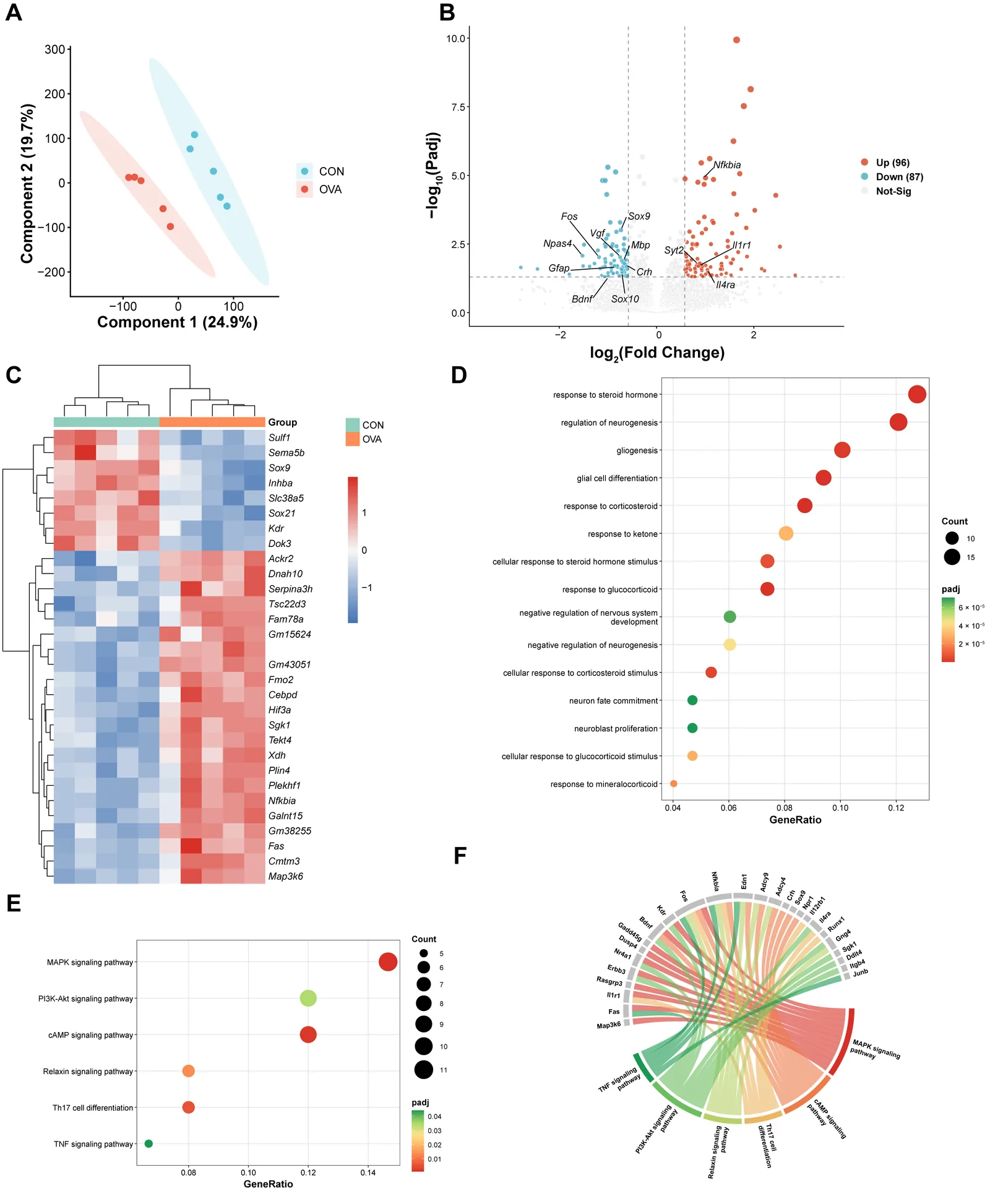
Transcriptomic remodeling and functional enrichment analysis of the PFC in OVA-induced food allergy. (**A**) PLS-DA score plot showing separation between the CON and OVA groups. (**B**) Volcano plot showing differentially expressed genes (DEGs) between the CON and OVA groups, including 96 upregulated and 87 downregulated genes. (**C**) Hierarchical clustering heatmap showing the expression patterns of selected DEGs across samples. (**D**) Gene Ontology (GO) enrichment analysis of DEGs. (**E**) Kyoto Encyclopedia of Genes and Genomes (KEGG) pathway enrichment analysis. (**F**) KEGG chord diagram illustrating the associations between selected DEGs and enriched pathways. *n* = 5/group. DEGs were defined by an adjusted *p* value < 0.05 and |log2 fold change| > 0.58.

To characterize molecular alterations in the PFC associated with food allergy, we performed transcriptomic sequencing of PFC tissue from OVA and CON mice (Figure 5). Partial least squares discriminant analysis (PLS-DA) showed clear separation between the two groups along the first (24.9% variance) and second (19.7% variance) components (Figure 5A), indicating distinct global transcriptional profiles. Differential expression analysis identified 183 DEGs in OVA mice relative to CON mice, including 96 upregulated and 87 downregulated genes (Figure 5B). Hierarchical clustering of selected DEGs further demonstrated distinct expression patterns between the two groups (Figure 5C).

The DEGs showed two prominent transcriptional patterns, characterized by upregulation of genes related to immune and inflammatory responses and downregulation of genes associated with neuronal activity and plasticity. Among the upregulated genes, *Map3k6*, *Cebpd*, *Il12rb1*, *Il4ra*, *Nfkbia*, and *Il1r1* are involved in cytokine responsiveness, inflammatory signaling, and stress-associated transcriptional regulation. In contrast, several genes associated with activity-dependent transcription, neurotrophic signaling, synaptic plasticity, and neuronal adaptation, including *Npas4*, *Fos*, *Bdnf*, *Vgf*, *Crh*, and *Junb*, were significantly downregulated. These gene-level changes indicate that food allergy-associated transcriptional alterations in the PFC were not restricted to immune-related responses but also involved molecular programs related to neuronal function.

GO enrichment analysis showed that the DEGs were enriched in biological processes related to central nervous system regulation, including regulation of neurogenesis, gliogenesis, and glial cell differentiation (Figure 5D). KEGG analysis identified several significantly enriched pathways, including MAPK, PI3K-Akt, cAMP, and TNF signaling, as well as Th17 cell differentiation (Figure 5E). The KEGG chord plot further illustrated associations between representative DEGs and enriched pathways, including the linkage of *Bdnf* and *Sgk1* to PI3K-Akt signaling (Figure 5F).

### 2.5. OVA-Induced Food Allergy Increases Relative Akt Ser473 Phosphorylation in the PFC

**Figure 6:**
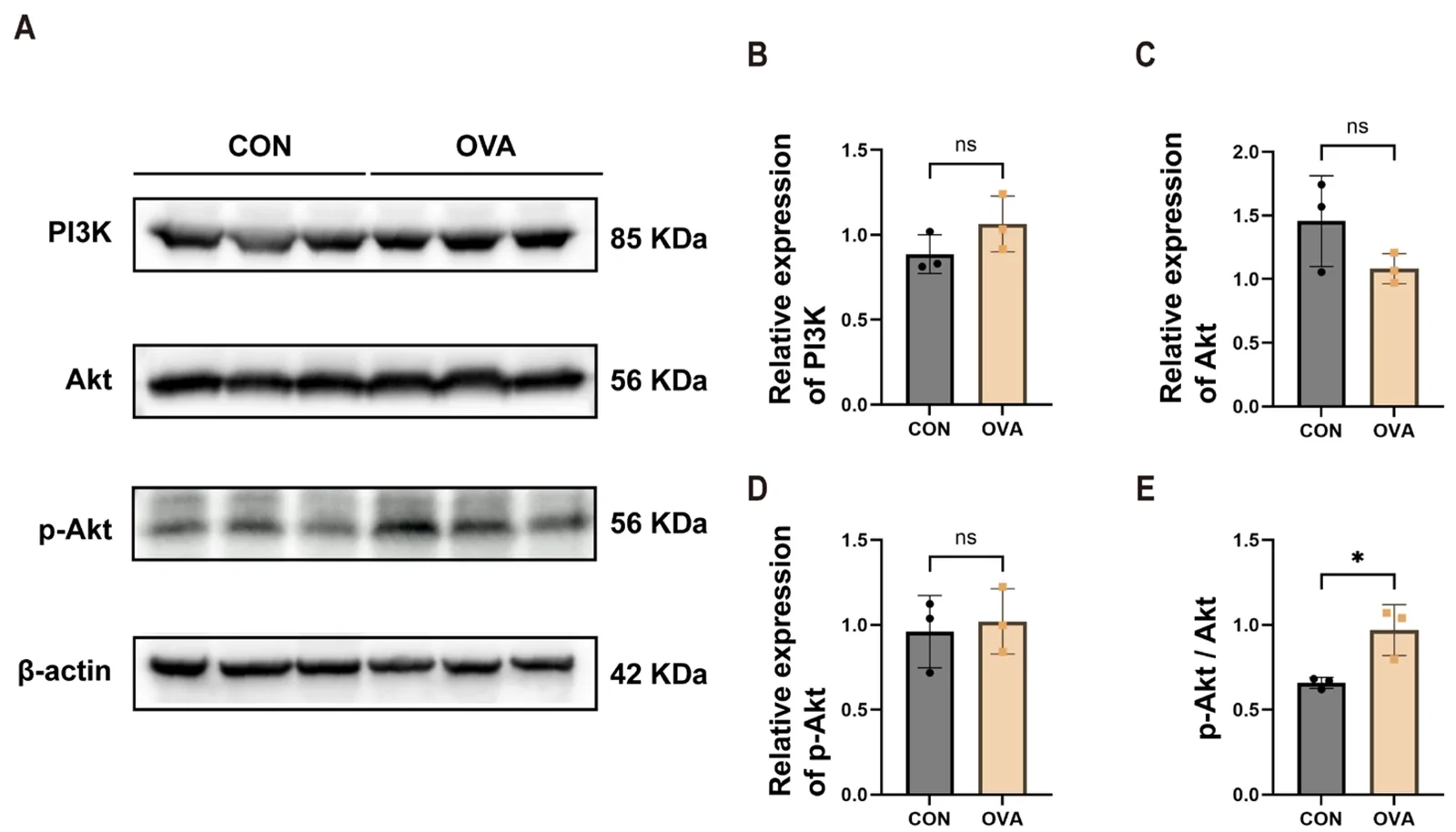
OVA-induced food allergy increases relative Akt Ser473 phosphorylation in the PFC. (**A**) Representative Western blot bands for PI3K, Akt, and p-Akt (Ser473) in PFC tissue from the CON and OVA groups. (**B**–**D**) Quantification of PI3K, total Akt, and p-Akt (Ser473) protein levels, respectively. (**E**) Ratio of p-Akt (Ser473) to total Akt. *n* = 3/group. Data are presented as mean ± SD. * *p* < 0.05; ns, not significant. CON, control; OVA, ovalbumin.

Given the significant enrichment of PI3K-Akt signaling and its association with representative dysregulated genes, including Bdnf and Sgk1, we further examined this pathway at the protein level by Western blotting (Figure 6A). PI3K protein expression did not differ significantly between groups (Figure 6B), and total Akt expression was likewise unchanged (Figure 6C). The absolute level of p-Akt (Ser473) also did not differ significantly between groups (Figure 6D).

However, the p-Akt (Ser473)/Akt ratio was significantly higher in OVA mice than in CON mice (Figure 6E), indicating increased relative phosphorylation of Akt at Ser473 without a marked change in total Akt abundance. These protein-level findings further support altered Akt-related signaling in the PFC identified by the transcriptomic analysis.

## 3. Discussion

Previous studies have increasingly linked food allergy to neurobehavioral abnormalities and neuroimmune changes in the brain [11,13], with PFC inflammation and microglial activation reported in OVA-induced food allergy [11]. However, the broader molecular alterations occurring in the PFC under food-allergic conditions remain insufficiently characterized. A key finding of the present study is that OVA-induced food allergy was associated with two directionally distinct transcriptional patterns in the PFC, characterized by upregulation of genes related to immune and inflammatory responses and downregulation of genes associated with neuronal activity and plasticity. These transcriptional changes occurred in parallel with increased local inflammatory and chemotactic mediators and microglial morphological remodeling, suggesting that PFC alterations in food allergy extend beyond a classical neuroinflammatory response and involve broader neuroimmune–neuronal molecular remodeling. In addition, PI3K-Akt signaling emerged from the transcriptomic analysis as a pathway potentially relevant to both neuroimmune regulation and neuronal function, and subsequent protein analysis showed increased relative Akt Ser473 phosphorylation. Together, these findings extend previous observations of PFC neuroinflammation by providing a transcriptome-level perspective on food allergy-associated brain alterations and suggest that immune-related and neuronal function-related molecular changes may occur concurrently in the PFC.

Food allergy-associated phenotypes can vary substantially across experimental models, with mouse strain, sex, allergen, and sensitization protocol contributing to differences in allergic and behavioral responses [20,21,22,23]. BALB/c mice are widely used in experimental food allergy, and a direct comparison with C3H/HeNCrl mice in a cow’s milk allergy model showed that BALB/c mice developed stronger humoral and Th2 responses, with higher levels of allergen-specific antibodies and Th2-associated cytokines [24]. Sex is another important biological variable. In an OVA-induced food allergy model, female BALB/c mice developed more severe systemic allergic responses and greater intestinal mucosal impairment than males [25]. Based on these observations, we selected 3-week-old female BALB/c mice, with sensitization initiated after a 1-week acclimation period. This design allowed allergen exposure to begin during the juvenile period and continue across an active stage of growth and development, with the aim of examining the effects of food allergy arising during this developmental window.

Within this model, OVA mice showed reduced exploration of open or exposed areas in both the OFT and EPM. Interpretation of the OFT requires caution because reduced center-zone exploration was accompanied by decreased total distance and velocity and increased immobility, suggesting that reduced spontaneous locomotion may partially contribute to the phenotype. However, OVA mice also showed reduced open-arm exploration in the EPM, whereas total arm entries remained unchanged, indicating that open-area avoidance could not be fully explained by generalized hypoactivity. Taken together, the convergent findings from the two behavioral paradigms support the presence of anxiety-like behavioral alterations in food-allergic mice, although additional behavioral tests and direct assessment of motor function would further strengthen this interpretation.

The behavioral alterations observed here should be interpreted in the context of the systemic consequences of food allergy. In addition to PFC changes, OVA mice exhibited systemic allergic responses, elevated circulating inflammatory mediators, and intestinal mucosal injury, all of which may influence behavioral performance. Importantly, systemic inflammation is not specific to food allergy. Studies of nonallergic inflammatory conditions have shown that peripheral immune activation can influence the brain and induce sickness-related behaviors, including reduced spontaneous activity and exploration, which may overlap with measures of anxiety-like behavior [26,27]. Thus, although the OFT and EPM findings support anxiety-like avoidance, systemic inflammation, intestinal injury, and other consequences of allergic reactions may also contribute to the observed phenotype. The present study cannot determine the relative contributions of these peripheral disturbances and PFC neuroimmune alterations to the behavioral changes.

The PFC is a key brain region involved in emotion regulation, threat appraisal, and anxiety-related behavior [14,15,16], and accumulating evidence has linked local PFC neuroinflammation to behavioral abnormalities [18,28]. In the present study, OVA-induced food allergy was associated with marked changes in the local PFC immune microenvironment. Levels of the pro-inflammatory cytokines IL-1β, TNF-α, IL-6, and IL-17A, as well as the chemokines CCL2 and CXCL1, were significantly increased. These mediators are involved in neuroinflammatory signaling, chemotactic responses, and immune–neural communication [29,30,31], suggesting that food allergy is associated with a broader disturbance of local neuroimmune homeostasis rather than isolated changes in individual inflammatory factors. Microglia, as resident immune cells of the CNS, continuously sense changes in the local microenvironment and undergo morphological and functional remodeling in response to inflammatory and stress-related signals [19,32,33,34]. Upon activation, microglia can release inflammatory mediators and modulate neuron–glia signaling, thereby influencing neuronal activity and synaptic homeostasis [19,35,36]. Consistent with an activation-associated response, Iba1 immunofluorescence showed a significant increase in microglial soma area in OVA mice, whereas the increase in Iba1-positive cell number did not reach statistical significance, indicating morphological remodeling rather than an increase in microglial abundance. Similar increases in PFC microglial soma area have also been reported in OVA-induced food allergy [11], further supporting the involvement of microglial alterations in the local PFC response to food allergy. Taken together, the concurrent changes in inflammatory and chemotactic mediators and microglial morphology indicate that food allergy is accompanied by substantial remodeling of the PFC neuroimmune microenvironment.

The present transcriptomic analysis further revealed marked changes in transcriptional programs related to immune and inflammatory responses in the PFC. Several upregulated DEGs, including *Il1r1*, *Il4ra*, *Il12rb1*, *Nfkbia*, *Cebpd*, and *Map3k6*, are involved in cytokine responsiveness, inflammatory signal transduction, and transcriptional regulation of immune responses. Among these genes, *Il1r1* encodes IL-1 receptor type 1, the ligand-binding receptor for IL-1α and IL-1β [37], and its upregulation occurred in parallel with the increased PFC levels of IL-1β, suggesting coordinated alterations in IL-1-related signaling. *Il4ra* encodes a key receptor subunit for IL-4 signaling and may reflect altered responsiveness to type 2 cytokine signaling [38]. *Il12rb1* encodes a receptor subunit shared by the IL-12 and IL-23 signaling complexes [39], suggesting broader remodeling of cytokine-responsive pathways. In addition, *Nfkbia*, *Cebpd*, and *Map3k6* are involved in inflammatory transcriptional regulation and stress-responsive signaling [40,41,42]. Consistent with these gene-level changes, KEGG analysis identified significant enrichment of TNF signaling, MAPK signaling, and Th17 cell differentiation. TNF signaling is a central regulator of inflammatory and immune responses [43], whereas MAPK signaling integrates inflammatory and cellular stress signals and can participate in both immune regulation and neuronal function [44]. The enrichment of Th17 cell differentiation further indicates that the transcriptional changes encompassed multiple immune-regulatory processes. Collectively, these findings extend the observed cytokine and microglial abnormalities to the transcriptomic level and demonstrate broader remodeling of cytokine-responsive and inflammatory programs in the PFC.

Importantly, the transcriptional alterations were not confined to immune-related processes. A distinct group of downregulated DEGs, including *Npas4*, *Bdnf*, *Fos*, *Junb*, *Crh*, and *Vgf*, is associated with activity-dependent transcription, neurotrophic signaling, synaptic plasticity, and neuronal adaptation [45,46,47,48,49,50,51,52]. NPAS4 is an activity-dependent transcription factor that regulates neuronal excitability, synaptic organization, and circuit plasticity [45,46,47], and alterations in NPAS4 expression in the PFC have been linked to synaptic and circuit adaptations after chronic stress [47]. BDNF is a key neurotrophic factor involved in neuronal function and synaptic plasticity, and dysregulation of BDNF has been implicated in affective disorders [49,50,51]. Neuroinflammation has also been proposed to influence BDNF signaling and synaptic plasticity through microglia-associated inflammatory processes, thereby contributing to anxiety-related behavior [49]. In addition, *Fos* and *Junb* are components of activity-dependent immediate early transcriptional responses involved in neuronal and synaptic adaptation [52]. GO enrichment further implicated biological processes related to CNS regulation, including regulation of neurogenesis, gliogenesis, and glial cell differentiation, indicating that the transcriptional changes extended to broader neural and glial programs. Collectively, these findings extend previous observations of PFC neuroinflammation in food allergy by revealing concurrent alterations in transcriptional programs related to neuronal activity and plasticity. However, representative DEGs were not directly validated at the protein level, and further studies are needed to determine whether these transcriptional changes are accompanied by corresponding alterations in protein expression.

Among the pathways identified by KEGG analysis, PI3K-Akt signaling was prioritized for further validation based on both the transcriptomic findings and its biological relevance. PI3K-Akt signaling was significantly enriched, and representative dysregulated genes, including *Bdnf* and *Sgk1*, were linked to this pathway in the chord analysis (Figure 5F). Moreover, PI3K-Akt signaling participates in microglial responses and inflammatory signaling as well as neuronal survival and synaptic plasticity [53,54,55,56], suggesting that this pathway may be relevant to both the immune-related and neuronal transcriptional alterations observed in the PFC. Alterations in PI3K-Akt signaling in the medial PFC (mPFC) have also been associated with anxiety-like behavioral changes in experimental models [57], suggesting potential relevance of this pathway to the behavioral phenotype observed in the present study. Western blot analysis showed no significant changes in total PI3K, total Akt, or absolute p-Akt (Ser473) levels, whereas the p-Akt (Ser473)/Akt ratio was significantly increased in OVA mice. These findings indicate an increase in the relative phosphorylation state of Akt rather than changes in total PI3K or Akt abundance. Previous studies have reported divergent changes in Akt phosphorylation across neuroinflammatory models. LPS-induced microglial activation has been associated with increased Akt phosphorylation [58], whereas reduced p-Akt levels have been reported in poly(I:C)-induced PFC neuroinflammation [59], highlighting the context-dependent regulation of Akt signaling. Together with the transcriptomic enrichment of PI3K-Akt signaling, the increased p-Akt/Akt ratio provides protein-level support for altered Akt-related signaling in the PFC. These findings further suggest that Akt-related signaling may represent a molecular link between the neuroimmune and neuronal alterations associated with food allergy. However, because no Akt-targeted intervention was performed, the causal role of Akt signaling in food allergy-associated neuroimmune and behavioral alterations remains to be determined.

The present study was conducted in female BALB/c mice using an OVA-induced food allergy model. Given the variability in allergic responses across species, strains, and sexes, the extent to which these findings can be extrapolated to human food allergy remains uncertain. Nevertheless, the PFC transcriptomic data can help prioritize genes with human orthologs and associated signaling pathways for cross-species comparisons and functional investigation. These findings also raise a clinically relevant question: whether allergy-associated immune changes are linked to emotional symptoms in patients with food allergy, while considering disease-related psychological burden. To build on these findings, future work could assess the reproducibility of these molecular alterations in other food allergy models and investigate their functional relevance to behavioral changes. Clinical studies integrating food allergy characteristics, peripheral immune measurements, and validated psychological assessments could then explore whether related immune–behavioral associations are present in humans.

Overall, the present findings indicate that OVA-induced food allergy in female BALB/c mice is associated with anxiety-like behavioral alterations, remodeling of the PFC neuroimmune microenvironment, and marked transcriptomic changes in the PFC. The present study extends current understanding of food allergy-associated brain alterations by showing that PFC changes are not limited to neuroinflammatory responses but also involve broader transcriptional remodeling related to neuronal activity and plasticity. The concurrent alteration of immune-related and neuronal transcriptional programs provides a molecular context for the anxiety-like behavioral phenotype observed in food-allergic mice. Altered Akt-related signaling may represent one molecular link between these neuroimmune and neuronal changes, providing a mechanistic framework for understanding the broader molecular consequences of food allergy in the PFC.

## 4. Materials and Methods

### 4.1. Animals

Three-week-old specific pathogen-free female BALB/c mice were purchased from Shanghai Ji-Hui Laboratory Animal Breeding Co., Ltd. (Shanghai, China). Mice were housed under standard barrier conditions at 22 ± 0.5 °C and 50% ± 10% relative humidity under a 12 h light/12 h dark cycle, with free access to standard chow and water. Mice were acclimated for 1 week before experimentation. All animal procedures were conducted in accordance with relevant animal welfare and ethical guidelines and were approved by the Institutional Animal Care and Use Committee of SCMC (Approval No.: SCMCIACUCPRS-K2019029).

### 4.2. Experimental Design

As illustrated in Figure 1, on Days 1, 8, and 15, mice in the OVA group received an intraperitoneal injection of 200 μL of sensitization solution containing 50 μg OVA (Sigma-Aldrich, Burlington, MA, USA; A5505) and 2 mg aluminum hydroxide adjuvant (Sigma-Aldrich, Burlington, MA, USA; 239186) in sterile saline. CON mice received an equal volume of sterile saline containing 2 mg aluminum hydroxide at the corresponding time points. Beginning on Day 22, OVA mice received 200 μL of OVA solution containing 50 mg OVA by intragastric gavage every other day, for five challenges on Days 22, 24, 26, 28, and 30. CON mice received an equal volume of sterile saline at the corresponding time points. Body weight was recorded at predefined time points throughout the experiment. After the final OVA challenge on Day 30, mice were observed continuously for 60 min, and anaphylactic responses were evaluated using a 0–5 scoring system according to the criteria shown in Table 1. Behavioral testing was performed on Days 32 (OFT) and 34 (EPM), followed by terminal blood and tissue collection on Day 36.

### 4.3. Behavioral Tests

Behavioral testing was conducted to evaluate the effects of food allergy on anxiety-like behavior. All behavioral experiments were performed in a quiet laboratory under consistent lighting and environmental conditions. Mice were allowed to acclimate to the testing environment before formal assessment. Movement trajectories were recorded using a video-tracking system and automatically analyzed using EthoVision XT 17 software (Noldus Information Technology, Wageningen, The Netherlands).

#### 4.3.1. Open Field Test

The open field test (OFT) was used to assess spontaneous locomotor activity, exploratory behavior, and anxiety-related behavior. The apparatus measured 40 cm × 40 cm × 50 cm, with the floor divided into a center zone and a peripheral zone. Mice were gently placed at a designated starting position and allowed to explore freely for 10 min, during which movement trajectories and activity states were recorded. Total distance traveled, velocity, center-zone time, and immobility time were quantified from the tracking data.

#### 4.3.2. Elevated Plus Maze

The elevated plus maze (EPM) was used to assess anxiety-like behavior. The apparatus consisted of two perpendicular open arms and two closed arms, positioned 80 cm above the floor. Both open and closed arms measured 30 cm × 6 cm, and the closed arms had 15-cm-high walls. Mice were placed in the center of the maze with their heads facing an open arm and allowed to explore freely for 5 min. Open-arm time, open-arm entries, open-arm entry percentage, and total arm entries were quantified from video-tracking data.

### 4.4. Immunological Analyses

#### 4.4.1. ELISA

On Day 36, mice were anesthetized by intraperitoneal pentobarbital administration combined with inhaled isoflurane, after which peripheral blood was collected, and the animals were euthanized. Blood was allowed to stand at room temperature for 2 h and centrifuged at 3500× *g* for 10 min at 4 °C to isolate serum. Serum was aliquoted and stored at −80 °C until analysis. Serum OVA-specific IgE (Cayman Chemical, Ann Arbor, MI, USA; 500840) and OVA-specific IgG1 (Cayman Chemical, Ann Arbor, MI, USA; 500830) were measured using ELISA kits according to the manufacturers’ instructions. Serum samples were diluted 1:50 for OVA-specific IgE and 1:128,000 for OVA-specific IgG1 measurements. Absorbance was measured after substrate development, and concentrations were calculated from standard curves.

#### 4.4.2. XMPlex Multiplex Cytokine Assay

Peripheral blood was collected on Day 36, and serum was prepared as described above. PFC tissue was collected on the same day, homogenized in protein lysis buffer containing a protease and phosphatase inhibitor mixture (Thermo Fisher Scientific, Waltham, MA, USA; 1861281), and centrifuged at 12,000× *g* for 20 min at 4 °C. Total protein concentration was determined using a BCA assay kit (Beyotime, Shanghai, China; P0010). Serum and PFC lysates were analyzed using an XMPlex multiplex assay (XMPlex02088; XMBIOTECH, Wuhan, China) on an XMPlex-100 analyzer according to the manufacturer’s instructions. Briefly, samples or standards (25 μL/well) were incubated with magnetic beads at 37 °C for 1 h, followed by incubation with detection antibodies for 30 min and a fluorescent reporter reagent for 15 min. Cytokine concentrations were calculated from standard curves.

### 4.5. Histopathological and Immunofluorescence Analyses

#### 4.5.1. H&E Staining

On Day 36, small-intestinal tissue was collected from the same anatomical location in each mouse. Tissues were fixed in 4% paraformaldehyde, dehydrated through graded ethanol, cleared in xylene, and embedded in paraffin. Paraffin sections were stained with hematoxylin and eosin (H&E), followed by dehydration, clearing, and mounting. Intestinal mucosal morphology was examined by light microscopy, and representative images were acquired.

#### 4.5.2. Immunofluorescence Staining

For immunofluorescence analysis, mice underwent transcardial perfusion with PBS, followed by 4% paraformaldehyde. Brains were collected and sectioned at a thickness of 10 μm. The PFC was identified according to the mouse brain atlas. Sections were blocked for 2 h at room temperature in PBS containing 10% goat serum (Absin, Shanghai, China; abs933) and 0.1% Triton X-100 (Sigma-Aldrich, Burlington, MA, USA; T8787), followed by overnight incubation with an anti-Iba1 monoclonal antibody (1:1000; Abcam, Cambridge, UK; ab178846). After thorough washing with PBS, sections were incubated with Alexa Fluor 594-conjugated secondary antibody (1:500; Thermo Fisher Scientific, Waltham, MA, USA; A150080). Nuclei were counterstained with DAPI, and sections were mounted. Fluorescence images were acquired using a Leica confocal microscope (Leica Microsystems, Wetzlar, Germany). Images were analyzed using ImageJ software (version 1.54p; National Institutes of Health, Bethesda, MD, USA). The number of Iba1-positive microglia and microglial soma area were quantified from the acquired images.

### 4.6. Transcriptomic Analysis

On Day 36, PFC tissues were collected from CON and OVA mice, and total RNA was extracted. RNA samples that passed quality control were subjected to bulk RNA sequencing on an Illumina NovaSeq X Plus platform (Illumina, San Diego, CA, USA). Sequencing data were quality-controlled and quantified, and differential expression analysis between the CON and OVA groups was performed using DESeq2 (version 1.48.1). Differentially expressed genes (DEGs) were defined as those with an adjusted *p* value < 0.05 and an absolute log2 fold change > 0.58. Partial least squares discriminant analysis (PLS-DA), volcano plots, and hierarchical clustering heatmaps were used to visualize sample distribution and DEG expression patterns. Gene Ontology (GO) and Kyoto Encyclopedia of Genes and Genomes (KEGG) enrichment analyses were performed in R (version 4.5.0) to characterize biological processes and signaling pathways associated with food allergy. The RNA-seq data generated in this study have been deposited in the NCBI Sequence Read Archive (SRA) under BioProject accession number PRJNA1515371, and SRA accession numbers SRR40267460–SRR40267469.

### 4.7. Western Blot Analysis

PFC tissues were homogenized in protein lysis buffer containing a protease and phosphatase inhibitor mixture (Thermo Fisher Scientific, Waltham, MA, USA; 1861281). Total protein concentrations were determined using a BCA assay kit (Beyotime, Shanghai, China; P0010). Equal amounts of protein were separated by SDS-PAGE and transferred onto PVDF membranes. After blocking, the membranes were incubated overnight at 4 °C with primary antibodies against PI3K (1:1000; 4257), Akt (1:1000; 4691), p-Akt (Ser473) (1:1000; 4060), and β-actin (1:2000; 3700), all from Cell Signaling Technology (Danvers, MA, USA). After washing, the membranes were incubated with HRP-conjugated anti-rabbit IgG (1:2000; 7074) or anti-mouse IgG (1:2000; 7076), also from Cell Signaling Technology, as appropriate. Protein bands were visualized using an enhanced chemiluminescence detection system, and band intensities were quantified using ImageJ software (version 1.54p; National Institutes of Health, Bethesda, MD, USA). PI3K, Akt, and p-Akt (Ser473) levels were normalized to β-actin, and relative Akt phosphorylation is expressed as the p-Akt (Ser473)/Akt ratio.

### 4.8. Statistical Analysis

All statistical analyses were performed using GraphPad Prism 10.0. Data are presented as mean ± SD unless otherwise indicated. Body-weight data were analyzed using two-way repeated-measures analysis of variance (ANOVA). Anaphylactic scores were analyzed using the Mann–Whitney U test. Other comparisons between two groups were performed using two-sided unpaired Student’s t-tests, with Welch’s correction applied when variances were unequal. *p* < 0.05 was considered statistically significant.

## 5. Conclusions

Our findings demonstrate that OVA-induced food allergy in female BALB/c mice is accompanied not only by robust peripheral allergic responses and intestinal injury but also by anxiety-like behavior and marked neuroimmune alterations in the PFC. In OVA mice, increased PFC inflammatory and chemotactic signaling was accompanied by microglial morphological changes, while transcriptomic analysis revealed broader molecular remodeling characterized by upregulation of genes involved in immune and inflammatory responses and downregulation of genes associated with neuronal activity and plasticity. In addition, the p-Akt (Ser473)/Akt ratio was increased at the protein level, supporting altered Akt-related signaling in the PFC. Taken together, these findings provide a broader molecular characterization of PFC alterations associated with food allergy-associated anxiety-like behavior and suggest that altered Akt-related signaling may represent a molecular link between the neuroimmune and neuronal changes observed in the PFC.

## Figures and Tables

**Table 1:** Anaphylaxis scoring scales.

Score	Clinical Symptoms
0	No reaction/clinical symptoms
1	Scratching and rubbing around the nose and head
2	Puffiness around the eyes and mouth, pilar erecti, reduced activity
3	Wheezing, labored respiration and cyanosis around the mouth and tail
4	No activity after prodding or tremor and convulsion
5	Death

## Data Availability

The RNA-seq data generated in this study have been deposited in the NCBI Sequence Read Archive (SRA) under BioProject accession number PRJNA1515371 and SRA accession numbers SRR40267460–SRR40267469. The data are currently under embargo and will be publicly available upon release.

## Abbreviations

FA	Food allergy
OVA	Ovalbumin
PFC	Prefrontal cortex
OFT	Open field test
EPM	Elevated plus maze
H&E	Hematoxylin and eosin
IF	Immunofluorescence
PLS-DA	Partial least squares discriminant analysis
DEGs	Differentially expressed genes
GO	Gene Ontology
KEGG	Kyoto Encyclopedia of Genes and Genomes
CNS	central nervous system
LPS	Lipopolysaccharide
i.p.	Intraperitoneal
i.g.	Intragastric
ELISA	Enzyme-linked immunosorbent assay
PBS	Phosphate-buffered saline
BCA	Bicinchoninic acid assay
